# Bacterial biofilm under flow: First a physical struggle to stay, then a matter of breathing

**DOI:** 10.1371/journal.pone.0175197

**Published:** 2017-04-12

**Authors:** Philippe Thomen, Jérôme Robert, Amaury Monmeyran, Anne-Florence Bitbol, Carine Douarche, Nelly Henry

**Affiliations:** 1 Sorbonne Universités, UPMC Univ Paris 06 & CNRS, UMR 8237, Laboratoire Jean Perrin, Paris, France; 2 Université Paris Sud, UMR 8502, Laboratoire de Physique des Solides, Orsay, France; Technion Israel Institute of Technology, ISRAEL

## Abstract

Bacterial communities attached to surfaces under fluid flow represent a widespread lifestyle of the microbial world. Through shear stress generation and molecular transport regulation, hydrodynamics conveys effects that are very different by nature but strongly coupled. To decipher the influence of these levers on bacterial biofilms immersed in moving fluids, we quantitatively and simultaneously investigated physicochemical and biological properties of the biofilm. We designed a millifluidic setup allowing to control hydrodynamic conditions and to monitor biofilm development in real time using microscope imaging. We also conducted a transcriptomic analysis to detect a potential physiological response to hydrodynamics. We discovered that a threshold value of shear stress determined biofilm settlement, with sub-piconewton forces sufficient to prevent biofilm initiation. As a consequence, distinct hydrodynamic conditions, which set spatial distribution of shear stress, promoted distinct colonization patterns with consequences on the growth mode. However, no direct impact of mechanical forces on biofilm growth rate was observed. Consistently, no mechanosensing gene emerged from our differential transcriptomic analysis comparing distinct hydrodynamic conditions. Instead, we found that hydrodynamic molecular transport crucially impacts biofilm growth by controlling oxygen availability. Our results shed light on biofilm response to hydrodynamics and open new avenues to achieve informed design of fluidic setups for investigating, engineering or fighting adherent communities.

## Introduction

Bacterial communities living attached to surfaces represent a widespread lifestyle of the microbial world[1–4]. In nature, including human hosts, and in artificial environments, these three-dimensional structures are often immersed in an aqueous fluid in motion and subjected to hydrodynamic forces[2]. Besides, the advances of microfluidics are making inroads in several fields of microbiology, providing new tools to investigate processes developing under flow, such as bacterial biofilm formation. Multiple types of micro-fabricated channels with length scales ranging from micrometers to centimeters are now used[5–7]. This opens the way to gaining deeper insight into how hydrodynamics interacts with biofilm development. This knowledge is currently scattered throughout a large variety of interests, ranging from engineering concerns[8–10] to fundamental biological questions regarding gene regulation by shear flow[11]. Basically, strong-flow regimes have been studied for their role in biofilm detachment and deformation, revealing complex effects arising from the interdependence of the flow strength and the biofilm visco-elastic properties, but essentially showing physical responses to physical stress[10, 12–16]. In the milder regime of laminar flow with small Reynolds number, which includes shear stress values relevant for many real-life situations—e.g. vasculature of living hosts, quiet river flows, medical devices[17–19]—the spectrum of flow effects on biofilm development is also broad. It includes initial attachment control[20–23], quorum sensing regulation[24, 25], morphological changes[20], growth rate modification[26–29], metabolic switching[28], alteration of visco-elasticity[30, 31], variation of extracellular matrix production, and possibly gene expression modification[11, 32]. Some of these effects have clearly been shown to proceed by advection-diffusion mechanisms, e.g. [24] but others motivate the hypothesis of a biological response to mechanical cues[11, 32]. Indeed, hydrodynamics simultaneously controls some closely inter-related effects that are very distinct in nature—namely, molecular transport and mechanical stress. Generally, it is not known whether the observed phenotypic changes are driven by the alteration of the molecular distribution of a key compound or by the response of a signaling cascade to mechano-sensing. While mechano-transduction has long been recognized as an integral part of the cell signaling arsenal in eukaryotes[33, 34], responses to mechanical forces have received less attention in bacteria, except regarding cell response to osmotic shocks through the mechano-sensitive ion channel Msc, which is directly stretched open when the membrane is under turgor pressure[35]. Yet, recently, together with an increasing interest in micro-organisms on surfaces, attention has emerged towards how bacteria respond to mechanical cues, revealing a direct response of attached cells to mechanical stress applied by shear flow, at the level of gene expression[36–38]. To the best of our knowledge, such a causal relationship linking mechanical force sensing to gene expression has been clearly established only at the single-cell level, and not within a three-dimensional biofilm, where it remains a mere working hypothesis.

In this work, we study the driving forces underpinning the effects of hydrodynamics on a growing model biofilm. We aim in particular at unraveling the mechanical contributions from the solute transport ones. We worked in an *Escherichia coli* strain over-expressing the conjugation pilus F, known to promote biofilm formation. We designed a set of 5 micro-fabricated channels of variable height to grow the biofilm under continuous nutrient flow and explore a two-orders of magnitude range of shear stress values from a few tenths to a few tens of millipascals. The hydrodynamics of the setup was characterized using experimental and theoretical data. Mounted on the stage of a microscope, the device enabled continuous monitoring of channel colonization over the first 20 hours of biofilm development. Thereby, initial colonization patterns and three-dimensional growth kinetics could be determined over the whole range of shear stress values, unveiling a threshold value of shear stress which determined biofilm settlement.

Next, to investigate a potential biofilm response to hydrodynamics at the gene expression level, we performed a differential transcriptomic analysis of the cells coming from two biofilms grown under different shear stress regimes.

Our results show that mechanical stress precisely determines initial settlement pattern, orienting biofilm development characteristics with notable consequences on expansion kinetics. In contrast, we do not find any signature of mechano-sensing-related signaling upon mechanical stress reinforcement in the two biofilms studied. Instead, we show that, under conditions where no nutrient depletion occurs, the primary biological response of biofilms to distinct hydrodynamic conditions consists in a modulation of the low-O_2_ stress response. Incidentally, our results point out the ability of bacterial biofilms to initiate in low shear stress zones and strategically spread from these bases towards high shear stress areas—a feature of interest in the perspective both of controlling beneficial or deleterious adherent communities and of designing novel fluidic circuits.

In conclusion, our results clarify the question of how hydrodynamics impacts biofilm development: first, it physically controls initial settlement, next it influences biological response, involving not mechanotransduction but O_2_ distribution.

## Materials and methods

### Bacterial strains and culture conditions

The *Escherichia coli* strain used in this work is TG1-F’ a K12 –MG 1655 derivative carrying the plasmid F’tet, a gift from the Ghigo lab [39]. All cultures were performed from LB agar plate colonies grown overnight in LB medium at 37°C in an agitated Erlenmeyer flask in the presence of 7.5 μg/ml tetracycline, then diluted in M63B1 minimum medium supplemented with 0.4% glucose to provide an exponentially growing culture with an OD_600_ equal to 0.2 after a few hours incubation at 37°C.

### Microfabrication and biofilm growth

#### Millifludidic device

We micro-fabricated millifluidic channels 30 mm in length, 1 mm in width and with heights ranging from 250 μm to 1 mm. A polydimethylsiloxane (PDMS) mixture (RTV615A+B from Momentive) was poured at ambient temperature in a polyvinyl chloride home-micromachined mold and left to cure at least 3 hours in an oven set at 65°C. Then, the recovered templates were drilled for further plugging of adapted connectors and tubings. PDMS templates and glass coverslips were then cleaned using an oxygen plasma cleaner (Harrick) and immediately bound together to seal the channels. The last step consisted in adapting connections: we used stainless steel connectors (0.013" ID and 0.025" OD) and microbore Tygon tubing (0.020" ID and 0.06" OD) supplied by Phymep (France). The thin metallic connectors accommodate on the flow circuit a bottleneck which prevented upstream colonization. Next the plugged device—usually a set of five channels 250 μm; 350 μm; 500 μm; 750 μm and 1 mm in height, respectively—was fixed on the microscope stage using a customized holder. The medium was pushed into the channels at a controlled rate using syringe pumps.

#### Biofilm growth

The same amounts—approx. 9.10^5^ cells—of exponentially growing cells with an optical density at 600 nm equal to 0.2 were injected in the channels and allowed to settle down for 1h30 before starting medium flow (at t = 0) and imaging bacterial development in the channel. The whole experiment was thermostated at 37°C.

### Microscopy

#### Microscope

We used an inverted NIKON TE300 microscope equipped with motorized x, y, z displacements and shutters. Images were collected using a 20x S plan Fluor objective, NA 0.45 WD 8,2–6,9. Bright field images were collected in direct illumination (no phase). Fluorescence acquisitions were performed using an m-cherry dedicated filter configuration (Exc- FF01-575/25, DM pinkel FF436/514/604-Di01-25x36, Em. FF01-457/530/628-25 Semrock bright Line).

#### Image acquisition

We used a Hamamatsu ORCA-R2 EMCCD camera for time-lapse acquisitions of 1344x1024 pixels images with 12 bits gray level depth (4096 gray levels) and captured an *xy* field of view of 330 μm x430 μm. Bright field and fluorescence images were collected using 10 ms and 40 ms acquisition times, respectively. Unless otherwise stated, biofilms were imaged for 24 hours at the frequency of 30 frames per hour.

#### Image analysis

Image intensity per pixel averaged on defined regions of interest (ROIs) was collected using the NIKON proprietary software NIS. The data sheets edited by NIS were next exported to Matlab for further analysis of the biofilm development kinetics and growth parameters determination.

#### Signal calibration

To quantitatively monitor biofilm growth, we used the microscope as a microphotometer and defined a microscopic absorbance *A*_μ_ = ln (*I*_*0*_/*I*), where *I*_*0*_ is the intensity (averaged gray level per pixel) of images recorded on a channel filled with water and *I* the intensity of the channel containing the growing biofilm, the presence of which attenuates incident light transmission. The attenuation was related to biomass in the channel by analogy with the empirical Beer-Lambert law stating that light absorbance by a material is directly proportional to the sample thickness and concentration. We verified this by growing a series of 10 identical biofilms in parallel in 1 mm-height channels under 1 ml/h medium flow rate. At different time points along the biofilm growth, biofilm growth was stopped by arresting nutrient flow and immediately extracting the biofilm from the channel using repeated air jet pulses. The extracted material was then entirely resuspended in 300 μL of minimum medium by gentle pipetting. Next, the concentrated suspension was diluted in minimal medium for optical density (OD) measurements in a spectrophotomer cuvette, and biomass was evaluated considering an OD-specific concentration of *E*. *coli* cells in a suspension (i.e. the number of cells per milliliter at an OD of 1 measured at 600 nm) equal to 5.10^8^ cells/mL. We then obtained the relation between the amount of cells in the channel and the microscopic absorbance, *A*_μ_. For further evaluation of local biomass, we converted it into a relation linking *A*_μ_ and a cell surfacic concentration, *C*_*S*_, defined as the number of cells settling over a 1 mm^2^ channel surface: *C*_*S*_ = 3x10^7^ *A*_μ_. The details given in supplementary information (S1 Fig) also show that, as in the macroscopic version of the Beer-Lambert law, the absorbance-biomass relation deviates from the linear regime at high concentrations. In the conditions of *I*_*0*_ used for biofilm growth monitoring, the biomass detection signal started to level off for microscopic absorbance values equal to 0.7. Such nonlinearities were accounted for in the growth curve analysis.

### Velocity field measurement and calculation

Growth medium supplemented with red (580/605) fluorescent particles 1 μm in diameter (Molec. Probes, F-8821) was pushed into the channel at a defined flow rate. Then, *z*-stack images were recorded using an acquisition time (t_acq_) of 40 ms, which caused the particles to appear on the images as linear trails, the length of which yielded the local velocity *v*(*x*,*y*,*z*) in the channel through *v*(*x*,*y*,*z*) = *l(x*,*y*,*z)*/t_acq_, where *l*(*x*,*y*,*z*) is the length of the trail (once the bead diameter is subtracted). The experimental velocity fields were derived from the collection of trail lengths in all available (*x*,*y*,*z*)-positions in the z-stacks (S2 Fig).

Besides, we expressed the theoretical velocity field using the formula for a steady viscous flow of an incompressible fluid in a rectangular channel at low Reynolds number[40, 41]. This velocity field satisfies the Stokes equation with non-slip boundary conditions on the channel walls (at *y* = 0, *y* = *w* the channel width, *z* = 0 and *z* = *h* the channel height, for all *x*), and with an imposed pressure gradient *dp/dx* along the *x* direction. It reads^39^:

$$\frac{v\left(y,z\right)}{4v_{1}}=\frac{y}{w}\left(1-\frac{y}{w}\right)+\frac{z}{h}\left(1-\frac{z}{h}\right)-{\sum_{n=0}^{\infty }{\left(-1\right)}^{n}\left(\frac{2}{\left(2n+1\right)\pi }\right)}^{3}[\frac{\cosh\left(p_{n}\left(y-\frac{w}{2}\right)\right)}{\cosh\left(p_{n}\frac{w}{2}\right)}\cos\left(p_{n}\left(z-\frac{h}{2}\right)\right)+\frac{\cosh\left(q_{n}\left(z-\frac{h}{2}\right)\right)}{\cosh\left(q_{n}\frac{h}{2}\right)}\cos\left(q_{n}\left(y-\frac{w}{2}\right)\right)],$$ (1)

with $p_{n}=\frac{\left(2n+1\right)\pi }{h}$, $q_{n}=\frac{\left(2n+1\right)\pi }{w}$ and $v_{1}=v\left(\frac{w}{2},\frac{h}{2}\right)=\frac{-1}{8\eta }\frac{dp}{dx}\frac{w^{2}h^{2}}{w^{2}+h^{2}}$, where η is the dynamic viscosity of the fluid.

The associated volumetric flow rate *Q*, which can be obtained by integrating the velocity field over a transverse section of the channel, reads:

$$Q=4whv_{1}\left\{\frac{1}{3}-\sum_{n=0}^{\infty }{\left(\frac{2}{(2n+1)\pi }\right)}^{5}\left[\frac{h}{w}\tanh(p_{n}\frac{w}{2})+\frac{w}{h}\tanh(q_{n}\frac{h}{2})\right]\right\}.$$

The experimentally-measured velocity fields were adjusted to these theoretical expressions using Matlab's nonlinear least-square fitting algorithms (*lsqcurvefit* and *nlinfit*). We checked that the infinite series in the formulas for *v(y*,*z)* and *Q* converge quite fast, as can be guessed by the 1/(2*n*+1)^3^ and 1/(2*n*+1)^5^ dependences of the summed terms. In practice, the results of our fits varied by less than 0.1% upon cutting the series at *n* = 3 versus *n* = 2, and we also checked that no larger change occurred in the fitted parameters when including terms up to *n* = 10. Here, we always used results obtained by cutting the series at *n* = 3. The shear stress distribution was obtained by differentiating these velocity fields.

### RNA preparation

Total RNA was prepared from exponentially growing (OD_600_ ≈ 0.3–0.5)—called sample E—and saturated (OD_600_ ≈2.6–4.5)—called sample S—liquid cultures in M63B1 medium supplemented with 0.4% of glucose, and from biofilms grown in micro-channels under the strongest confinement considered, i.e. 250 μm-height (sample H) and under the lowest confinement considered, 1 mm-height (sample L). Biofilms were extracted from the channels by repeated air jet pulses and the cells immediately resuspended at 4°C as concentrated viscous aliquots of 200–300 μl. The OD was measured and an adequate volume was taken and centrifuged to get a pellet of approx. 10^9^ cells. All RNA was then isolated and purified with RiboPure-Bacteria Kit (Ambion, Life Technologies). Sample quality was checked and quantified by gel electrophoresis using Bioanalyser (Agilent); about 10 μg total RNA per sample was obtained. It was immediately stored at -80°C for time periods of maximum 5 weeks, prior to mRNA purification and RNA-Seq analysis. All samples were run in triplicates.

### RNA sequencing and data analysis

Sequencing was carried out using an Illumina HiSeq platform via a commercial service (Eurofins MWG GmbH, Ebersberg, Germany). Mapping of reads to reference sequences was performed using BWA-MEM (version 0.7.12-r1039, http://bio-bwa.sourceforge.net/). Raw read counts were created using featureCounts[42]. A Trimmed Mean of M-values normalization and differential expression analysis was performed using the edgeR package[43, 44]. Among all the twelve triplicated samples, one, which exhibited unrelated data, was removed from the analysis.

### Oxygen measurement

Dissolved O_2_ was measured by quenching a Ruthenium complex (Ruthenium–tris(4,7– diphenyl–1,10-phenanthroline) dichloride (Ru(dpp)); Fluka) encapsulated in micelles of (1,2–Dipalmitoyl–sn–Glycero–3–Phosphoethanolamine–N[Methoxy(Poly–ethylene glycol)–2000] (DMPC–PEG2000) phospholipids; Avanti. The encapsulation of the Ru(dpp) complex in the micelles[45] yields it biocompatible while allowing to exhibit a linear fluorescence response to the oxygen concentration. This response follows a Stern-Volmer plot that we further used to convert the fluorescence intensity into oxygen concentration[46]. Ru–micelles were added to the channels with or without biofilm at a concentration of 8 μM. Fluorescence images were collected using a 60x plan Apo Oil objective, NA 1.4 WD 0.21, and using an FITC dedicated filter configuration (FF01-482/35-25 FF506-DiO3-25x36 536/40-25). Images were acquired using 100 ms acquisition time. In order to get the fluorescence intensity value at 20% oxygen, a calibration experiment was performed by measuring the fluorescence of a solution saturated with 20% oxygen by vigorous shaking for several minutes.

## Results

### In a millifluidic channel, cells are exposed to a distribution of different shear stress values

To capture and understand the impact of fluid dynamics on biofilm formation features, we microfabricated a set of five channels with the same width, *w* (1 mm), and length (3 cm), but differing by their height, *h*, ranging from 250 μm to 1 mm.

**Fig 1:**
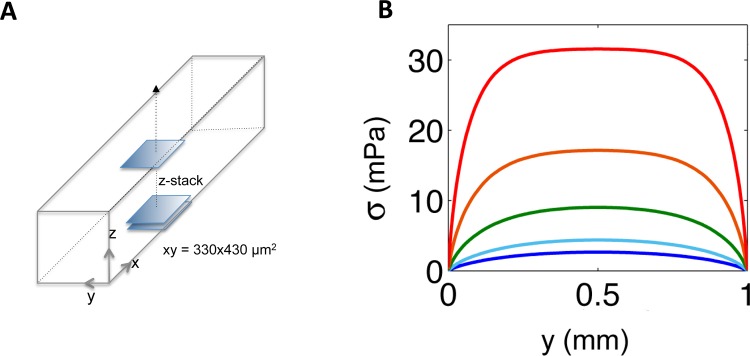
Shear stress significantly varies across the width of a millifluidic channel. (A) Channel blueprint. (B) Bottom shear stress values, obtained by differentiation of the calculated velocity fields, are given for the 5 PDMS channels having a 30 mm length, 1 mm width (*y* axis) and 250 μm (red); 350 μm (orange); 500 μm (green); 750 μm (light blue); 1 mm (dark blue) height, under a flow rate of 1 ml/h.

In order to clarify the hydrodynamic properties of our setup, we calculated the shear stress distribution profile in each channel, using the theoretical approach of O’Brien[40] to take into account their low-aspect ratio geometries, i.e. *w≈h* (see S3 Fig and Materials and Methods). Fig 1 shows that the 5 channels, under a flow rate, *Q*, of 1 ml/h (as in the rest of our work), exhibited shear stress values σ at the channel bottom (*z* = 0) spanning almost two orders of magnitude, from 0.42 to 31.5 mPa, depending on the considered channel and on the position with respect to the edge of the channel. The fluid velocity fields calculated using this theoretical framework were consistent with those derived from the experiments where we measured the length of fluorescent particle trails (S2 and S3 Figs). This agreement enabled us to derive the actual value of σ at any point of any channel knowing *h*, *w* and *Q*. The 1 ml/h flow rate was chosen to achieve biofilm installation within a few hours, while applying shear stresses relevant to physiological and natural situations[17–19]. This flow rate also guaranteed that glucose—the limiting nutrient—was always supplied in excess, so that no nutrient depletion occurred (S1 File).

### Fluid dynamics impacts initial colonization pattern and development kinetics

To initiate biofilm growth in the five channels mounted in parallel, we inoculated the same amount of bacteria (approx. 9.10^5^ cells) in each. After 1h30, growth medium flow was started and colonization was monitored by time-lapse video microscopy. Due to bacteria sedimentation during incubation, initial colonization occurred only on the bottom glass slide of the channel.

**Fig 2:**
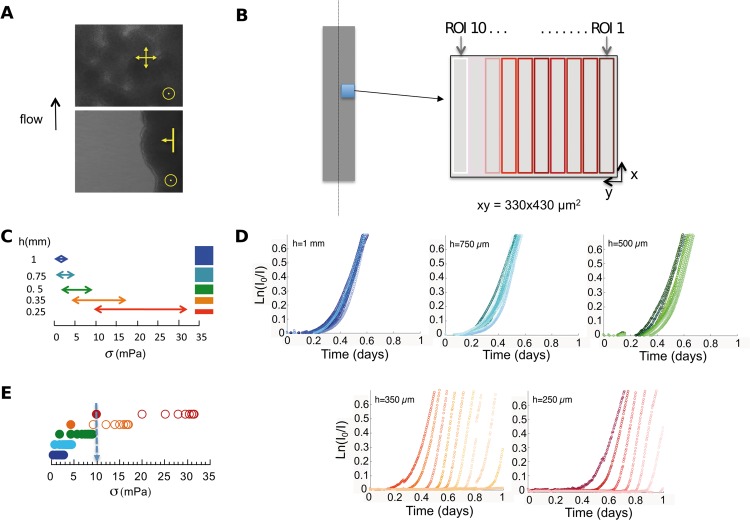
Quantitative monitoring of biofilm growth reveals shear stress impact on biofilm development. (A) Surface pattern of biofilm growing in the uniform growth mode (upper panel) and in the advancing-front growth mode (lower panel), yellow arrows indicate biofilm expansion directions; bright field picture taken using a 20x objective after 12 hours of continuous growth in M63B1 glucose medium in a 1 mm-height channel (uniform mode, upper panel) and a 250 μm-height channel (advancing-front mode, lower panel). The right edge of the picture coincides with the right edge of the channel, and a 330x440 μm^2^ field is imaged. (B) Images are divided in 10 adjacent ROIs of 40 μm width, numbered from 1 (darker shade) to 10 (lighter shade); ROI1 is the channel edge ROI and ROI10 is a central ROI, located at 400 μm from the channel edge. (C) Bottom shear stress ranges overlapping between the 5 channels of the setup with channel heights on the left and aspect ratios on the right. Same channel color code as in Fig 1. (D) Series of growth curves derived from time-lapse image analysis. The microscopic absorbance, *A*_μ_ = ln(*I*_*0*_/*I*), reporting the local biomass, is plotted as a function of time under continuous nutrient flow. Data points are represented in color for 10 ROIs per channel (one channel per graph) using the same color code as in Fig 1, and shades from dark to light coding for ROI1 to ROI10. Plot of a representative experiment of at least three (see data statistical dispersion in S1 Table). (E) Biofilm initiation is subjected to a 10 mPa shear stress threshold. Colonized ROIs (closed symbols) and non-colonized ones (open symbols), as observed in the 5 channels (same color code as in Fig 1) during the initial phase of biofilm development, are ordered on a shear stress range, evidencing the 10 mPa transition (arrow) between a shear stress which permits initial adhesion and a shear stress which does not.

Our first qualitative observations revealed two different development patterns. The conditions applied in the 3 channels imposing weakest confinement (height between 1 mm and 500 μm)—corresponding to the lowest shear stress regime—promoted a uniform development of the biofilm over the bottom surface of the channel. In contrast, in the geometries imposing higher shear stress and confinement, i.e. in the 250- and 350-μm-height channels, the biofilm developed according to an advancing-front mode, progressing from the edge towards the center of the channel (Fig 2A, S1–S5 videos).

To take into account the spatial distribution of the fluid dynamics within a given channel, we defined a set of ten rectangular regions of interest (ROIs), successively drawn along the *y*-axis of the field of view starting at the right edge of the image (see Fig 2B). Each ROI captured a channel segment characterized by a mean bottom shear stress which increased from the edge (ROI_1_) to the center of the channel (ROI_10_), with partially overlapping shear stress ranges in different channels (Fig 2C). Using calibration information (S1 Fig), the image stacks collected in each channel could be used to infer biomass expansion kinetics. Series of ten curves (one per ROI) reporting the time evolution of colonization of channel regions under different local hydrodynamic conditions were obtained. The 10-ROI-curve series exhibited either a bundle of curves closely grouped together on the time axis or a series of regularly spaced curves, consistently reflecting the two distinct—uniform and advancing-front—growth modes (Fig 2D).

Focusing on the initial period of colonization, i.e. the first 10 hours, we found that the 50 ROIs defined through the 5 channels could be sorted in two categories: those where bacteria could steadily attach to the surface within the first ten hours and directly initiate biofilm formation, and those where no initial attachment was observed. In the latter regions, space colonization took place through later expansion of the biofilm initiated in a low shear stress region of the channel near the edges, towards regions of higher shear stress values (see S4 and S5 videos). Characterizing each region by its mean bottom shear stress, we found a threshold shear stress value of 11±2 mPa, above which no direct initiation of biofilm could occur on the surface (Fig 2E and S5 Fig).

This initial obstacle to settlement favored the advancing-front growth mode for biofilms growing in the highest-confinement channels. Biofilm then expanded from the channel edges, where shear stress values are initially below 10 mPa. By contrast, uniform initial adhesion promoted uniform growth in the lowest-confinement channels featuring shear stress values below 10mPa throughout the channel.

**Fig 3:**
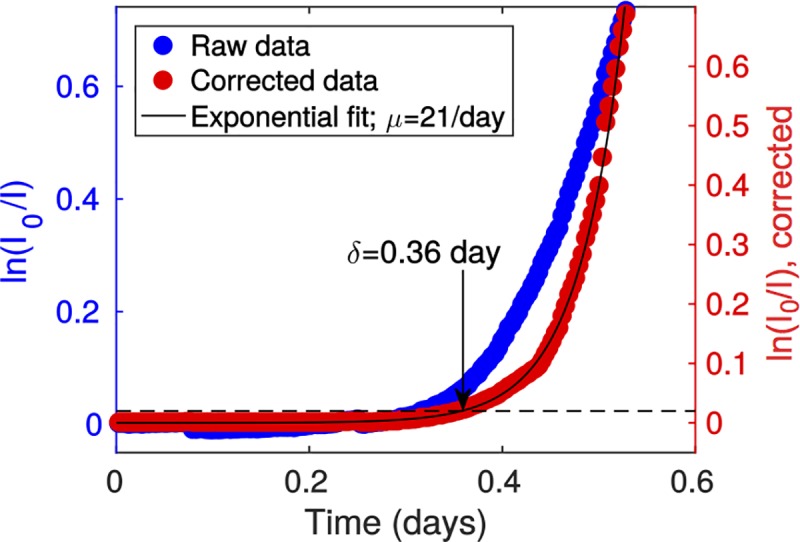
Exponential adjustment and derivation of growth parameters. A typical experimental curve showing microscopic absorbance (blue dots and left *y* axis) is represented together with the curve corrected by the microscopic absorbance-concentration relation in S1 Fig (red dots and right *y* axis) and the exponential adjustment (black line) giving the growth rate μ. The lag time δ is defined as the time where the corrected absorbance hits 0.02 (dashed line).

To find out how these two types of biofilms functionally differed, we determined specific growth parameters, namely the growth rate and the lag time, from the time evolution of the biomass, measured through the microscopic absorbance *A_μ_* = ln (*I*_*0*_/*I*) (see Fig 3 for an example). The measured absorbance was first corrected by the experimentally-measured relation between microscopic absorbance and concentration (see Fig 3 and calibration information in S1 Fig). This correction frees our analysis from the slight nonlinearities present even at low absorbance in this relation. We then performed exponential adjustments of the corrected absorbance curves *f(t)* (Fig 3), yielding the growth rate μ:

$$f(t)=f_{0}e^{\mu t}$$

We also considered the time δ at which the biomass reached a corrected absorbance value of 0.02, which provided an effective lag time for the colonization process (Fig 3). This threshold was chosen to be above experimental noise while remaining small, thus indicating growth onset.

**Fig 4:**
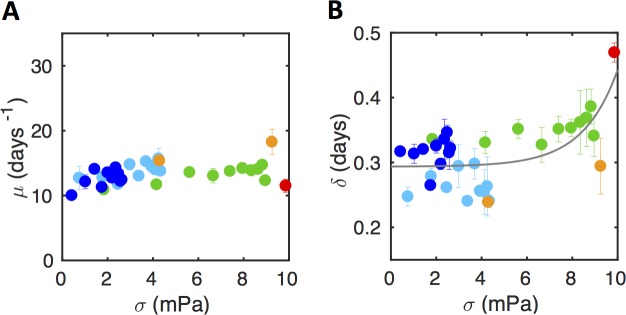
Below 10 mPa, shear stress does not affect biofilm growth rate, but impacts apparent lag time. (A) Biofilm growth rate, μ (as derived from the exponential adjustment, see Fig 3) as a function of bottom shear stress. (B) The apparent lag time, δ, increases as a function of shear stress; experimental data (solid colored dots) can be adjusted using an exponential law (grey line). Data were collected from biofilm growing in the low shear stress regime, i.e. at σ <10 mPa. Data are represented using the same channel color code as in Fig 1. Means and SDs over two positions are represented. Plot of a representative experiment of at least three (see data statistical dispersion in S1 Table).

Using this formalism, we first examined the case of mild shear stress regions (σ < 10 mPa). The results in Fig 4A show that the growth rate μ was constant both across the different positions in a given channel and across the different channels, showing that biofilm expansion rate was not sensitive to physical stress in these mild conditions. Besides, the apparent lag time δ increased with shear stress (correlation coefficient 0.55, p-value 0.001), indicating a shear stress-induced delay to initial cell docking (Fig 4B).

**Fig 5:**
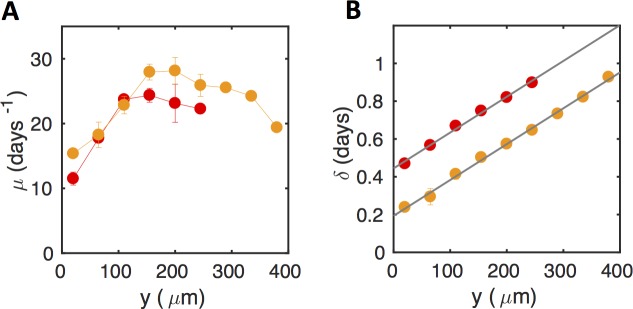
Local growth rate and lag time in the advancing-front growth mode characterize the biofilm spatial spreading. Biofilm growth rate, μ (A) as derived from the exponential adjustment, (see Fig 3) and apparent lag time δ (B), as a function of the lateral location in the channels in the high bottom shear stress regime (> 10 mPa)—the 250 μm-height one (red dots) and the 350 μm-height one (orange dots). The slope of the lag time linear adjustment (grey line) provides the front propagation velocity. Means and SDs over two positions are represented. Plot of a representative experiment of at least three (see data statistical dispersion in S1 Table).

Then, we explored the growth traits of the biofilms that developed under bottom shear stress values higher than 10 mPa, i.e. grown in the 250 μm- and 350 μm-height channels. Due to the advancing-front growth mode associated with this shear stress range, the expansion rate and lag time contained different information. Indeed, the increase of the biomass in a given ROI resulted from the material densification and vertical growth—as under mild shear stress—but also from a lateral directional input of mature material progressing from the edge. These combined contributions were indistinctly reported by the rate μ. In addition, in this growth mode, the apparent lag time δ(R_i_), associated with each ROI_*i*_ gave the time the front took to reach the ROI_*i*_ position, yielding the front velocity. Then, for all the colonized ROIs in the two high shear stress channels, we plotted μ and δ as a function of the distance *y* separating the right boundary of the considered ROI from the channel right edge (Fig 5). In both channels, we observed that lag times increased linearly with distance *y*, indicating a constant front velocity. In addition, both channels exhibited similar front velocities, given by the inverse of the slope of δ = f(*y*), equal to 529±10 μm/day and 513±10 μm/day for the 250 μm and 350 μm–height channels, respectively. The front progression delay between the two channels (0.22±0.05 day) was consistent with the lag time difference (0.16±0.05 day) expected from the shear stress values in their edge ROIs (4.3 mPa and 9.9 mPa in the 250 μm-height and 350 μm-height channels, respectively (Fig 4)).

Meanwhile, the growth rate, μ, increased with the distance to the edge of the channel up to y = 200 μm where it reached a maximal value (Fig 5). This indicates that, as the front pushed ahead, the biofilm also extended vertically. Both channels, despite differences in their confinement level (and thus in the shear stress applied to the developing material), exhibited similar colonization rates at the same location in the channel.

Interestingly, higher growth rates were measured in the biofilm growing under the advancing-front mode than in the uniform one (S2 Table), indicating a higher colonizing efficiency of the advancing growth mode in the phase of expansion of the biofilm.

These results show that the physical forces applied by fluid flow have a drastic impact on biofilm initiation on the surface, which indirectly impacts further biofilm colonization efficiency. However, they have no direct influence on biofilm intrinsic growth rate.

### Biofilm growth modulates hydrodynamics

**Fig 6:**
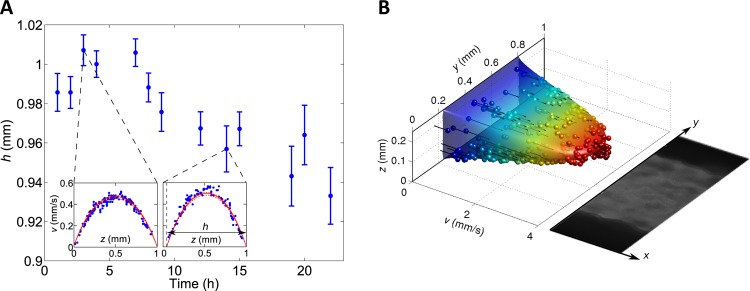
The velocity field evolves as the biofilm grows in the channel. **(A)** Effective height of a 1 mm-total-height channel in the presence of growing biofilm. Main panel: Adjusted effective height *h* versus time *t* of biofilm growth. Error bars represent 95% confidence intervals on *h*, obtained from the adjustment. Insets: examples of adjustment of the velocity field. Dots: experimentally-measured velocities *v* at various positions *(y*, *z)* across the channel. Data was collected for all *z* within a narrow band of *y* (width Δ*y* = 75 μm << *w*) located at the middle of the channel. Solid red curve: adjustment to the theoretical expression of the velocity field of a viscous fluid in a rectangular channel (Eq 1), plotted with *y* in the middle of the Δ*y*-wide band. Dashed red curves: idem for *y* at each edge of the Δ*y*-wide band. In addition to the effective height *h*, the volumetric flow rate *Q* was adjusted (through—η *dp/dx*), as in the absence of biofilm (S3 Fig), yielding *Q* = 0.85 mL/h, consistent with the adjusted value *Q* = 0.83 mL/h obtained in the biofilm-free case (S3 Fig). (B) Apparent size reduction in a 250 μm-height channel colonized by a biofilm after 15h growth. Dots: experimentally-measured velocities *v* at various positions *(y*, *z)* across the channel. Surface: adjustment to the theoretical expression of the velocity field of a viscous fluid in a rectangular channel (Eq 1). The color scale denotes velocities (see *v* axis). Black lines: distance between the experimentally-measured velocities and the adjusted values at the same location. Both the width *w* and the effective position *y*_0_ of the no-slip boundary condition in the *y* direction were adjusted, yielding *w* = 0.57 mm and *y*_0_ = 0.23 mm. The volumetric flow rate *Q* was set to its experimentally-imposed nominal value 1 mL/h, which yields a good adjustment in the biofilm-free case (S3 Fig). The coefficient of determination of the fit is R^2^ = 0.92. Picture: Growing biofilm snapshot showing the fluid (gray)/biofilm (black) interface matching the boundaries given by the fit.

Next, we wanted to examine to what extent biofilm growth affects the hydrodynamics in the channels. To this end, we first injected fluorescent beads in the fluid flow of the 1 mm-height channel while biofilm was growing. *z*-stack images were recorded at various stages of development to collect particle trails and derive the flow velocity fields. The data were adjusted using the theoretical formula of O’Brien (see Materials and Methods), leaving the effective channel height, *h*, as a free parameter derived from the fit. We plotted this effective height as a function of the biofilm growth time (Fig 6A). A similar approach was used to analyze the velocity field in the 250 μm-height channel in which biofilm had grown for 15 h, but this time, we took the channel width, *w*, as an adjustable parameter, in order to account for the advancing-front growth mode of the biofilm (Fig 6B). The quality of the adjustments of the flow velocity fields confirmed that, in the presence of the biofilm, flow characteristics corresponded to a conserved laminar flow in a rectangular channel, with a size reduced by an amount corresponding to the thickness of the biofilm.

Interestingly, the extrapolation to zero velocity corresponded to the biofilm/fluid interface, indicating that the fluid flow did not penetrate into the biofilm the surface of which thus defined the flow motion boundary.

From the velocity field characteristics, we calculated that in the 1 mm-height channel, the maximal bottom shear stress evolved from 2.67 mPa in the initial phase of growth (when biofilm thickness is negligible), to 3.02 mPa after 20 hours of growth (when a 70 μm thick biofilm layer has settled). Hence, the physical stress applied to the biofilm interface was not strongly affected (+13%). In contrast, in the 250 μm-height channel, the maximal bottom shear stress shifted from 31,5 mPa in the initial phase to 72,3 mPa after 15 h (when biofilm front has advanced 250 μm from the edge of the channel), demonstrating a significant increase of the bottom shear stress at the center of the channel. Yet, considering not this maximal bottom shear stress but the one at the biofilm advancing front, we notice that, within a 6 μm-wide strip adjoining the biofilm front, shear stress has values below 10 mPa. This region of low shear stress, resulting from the reshaping of the velocity field by biofilm growth, enables biofilm expansion in spaces that initially could not be colonized through direct adhesion to the channel bottom.

### Biofilms formed under different hydrodynamic regimes exhibit exquisitely distinct transcriptomes, pointing at the impact of O_2_ distribution

To investigate biological functions potentially associated with the uniform and advancing-front growth modes induced by the two shear stress regimes, we performed a whole transcriptome analysis of the two types of biofilm. We focused on samples extracted from the 250 μm-height channel, featuring the highest confinement and highest shear stress (sample H) and from the 1 mm-height channel, featuring the lowest confinement and lowest shear stress (sample L). The biofilms were extracted from the channels for RNA preparation in their maximal growth phase (see Materials and Methods). In addition, to confront the results with existing knowledge about biofilm-specific gene expression, we also studied cells harvested from planktonic exponentially growing (sample E) and saturated (sample S) cultures.

**Fig 7:**
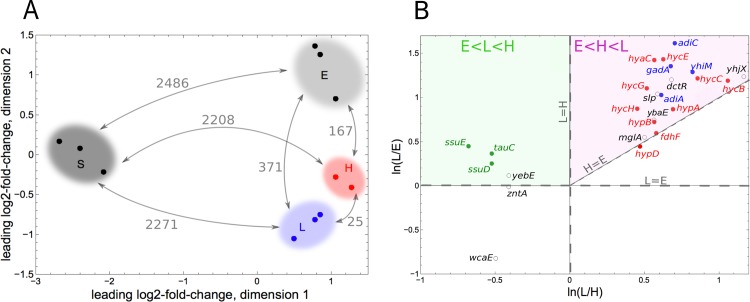
Biofilms under distinct fluid flow regimes exhibit exquisitely different gene expression. (A) Principal component analysis and clustering of genes differentially expressed in cells grown under different conditions: L (lower shear stress and lower confinement regime, i.e. 1 mm-height channel), H (higher shear stress and higher confinement, i.e. 250 μm-height channel), E (planktonic cells in exponential phase), S (planktonic cells in stationary phase). The number of genes with a False Discovery Rate < 0.01 in our differential expression analysis, is indicated above arrows. (B) 3-component plot representing ln(L/E) vs. ln(L/H) where L, E and H designate mean expression level in samples L, E and H respectively. Each point corresponds to a gene. Red: genes related to anaerobic metabolism; blue: genes related to acid resistance; green: genes related to sulfate metabolism; black: others genes. All red and blue genes (except *hypD*) fall in the purple zone: they have a lower expression in samples E and a higher expression in samples L, expression in samples H being intermediate.

Analyzing the transcriptome by principal component analysis showed that all the conditions could be clustered independently, with H and L biofilms appearing in neighboring regions of the multiscaling plot (Fig 7A). One of the sample H triplicates, which was inexplicably apart from the other samples (data not shown), was not taken into account in the differential expression analysis. Considering the pairwise distances between the four samples in number of genes exhibiting significantly differential expressions, i.e. a False Discovery Rate (FDR) <0.01, we found that 25 genes discriminated H and L samples, while the most different physiology was that of cells from saturated cultures (more than 2000 affected genes). Comparatively, only a few hundred genes were differentially expressed between exponentially growing planktonic cells and biofilm-dwelling cells, suggesting that cell populations of biofilms grown under continuous nutrient flow share more traits with exponentially growing cells than with stationary phase cells.

Further examination of the 25 genes differentially expressed in sample L versus sample H revealed that 19 were more expressed at low than at high shear stress (Table 1). Interestingly, 10 were associated with anaerobic metabolism[47] and 4 were linked to acidic conditions resistance. Besides, the expression levels of most of these genes were lower in samples E than in H (where they are lower than in L) (Fig 7B). In good agreement with this finding, these 14 genes have already been identified (or related to) genes involved in biofilm formation[48, 49]. Consistently, cytochrome oxidase genes were also differently expressed in H and L (S4 Fig).

**Table 1:** Genes most affected in expression in biofilms under flow: low fluid dynamical regime (L) versus high fluid dynamical regime (H). Second column is the ratio between mean normalized transcriptional expressions. Anaerobic metabolism genes (Bold), acid resistance genes (Italic), Sulfate metabolism genes (Underlined).

Genes with F.D.R. < 0.01	L/H^(a)^
yhjX, pyruvate-inducible inner membrane protein putative transporter	14,70
hycB, hydrogenase 3 Fe-S subunit	**11,44**
hycC, hydrogenase 3 membrane subunit	**7,18**
yhiM, acid resistance protein inner membrane	*6*,*65*
adiC, arginine:agmatine antiporter	*5*,*06*
hypA, protein involved in nickel insertion into hydrogenases 3	**4,92**
dctR, Putative LuxR family repressor for dicarboxylate transport	4,82
gadA, glutamate decarboxylase A PLP-dependent	*4*,*75*
hycE, hydrogenase 3 large subunit	**4,24**
adiA, arginine decarboxylase	*4*,*11*
slp, outer membrane lipoprotein	3,88
fdhF, formate dehydrogenase-H selenopolypeptide subunit	**3,80**
hypB, GTP hydrolase involved in nickel liganding into hydrogenases	**3,69**
hyaC, hydrogenase 1 b-type cytochrome subunit	**3,68**
ybaE, putative ABC transporter periplasmic binding protein	3,67
hycG, hydrogenase 3 and formate hydrogenase complex HycG subunit	**3,28**
mglA, methyl-galactoside ABC transporter ATPase	3,17
hypD, hydrogenase maturation protein	**2,96**
hycH, hydrogenase 3 maturation protein	**2,84**
zntA, zinc cobalt and lead efflux system	0,39
yebE, DUF533 family inner membrane protein	0,39
wcaE, putative glycosyl transferase	0,32
ssuD, alkanesulfonate monooxygenase FMNH(2)-dependent	0,30
tauC, taurine ABC transporter permease	0,30
ssuE, NAD(P)H-dependent FMN reductase	0,21

Besides, 6 genes were down-regulated by a factor 2.5 to 5 at low versus high fluid dynamics among which 3 were related to sulfate metabolism (Table 1).

All together, these results show that beyond the biofilm growth traits and developmental pattern, the major impact of the flow hydrodynamics on biofilm physiology regards aerobiosis regulation, showing enhanced low O_2_-stress level at low fluid flow.

### Biofilms under flow in a millifluidic channel grow under a low partial pressure of oxygen

As the transcriptomic analysis pointed to distinct levels of micro-aerobiosis under the two characteristic hydrodynamic conditions, we wanted to evaluate the direct impact of hydrodynamics on O_2_ supply in our setup. To assess oxygen supply in our specific configuration, we carried out an experimental determination of the partial pressure of O_2_ in the two channels, using Ruthenium micelle fluorescence.

**Fig 8:**
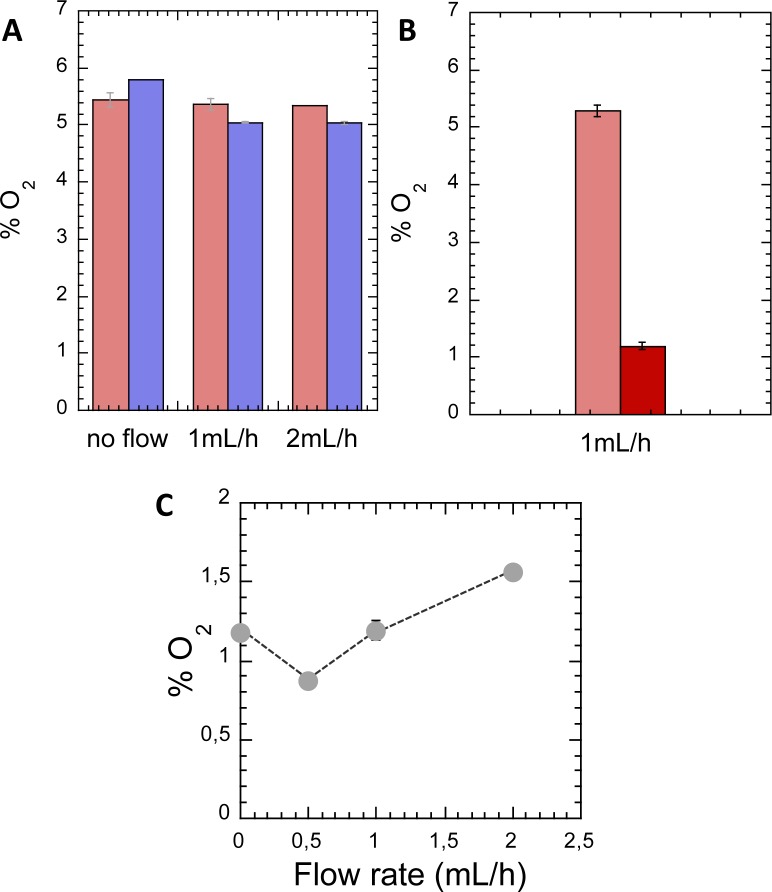
Biofilm grows at low O_2_ level in a millifluidic channel. Oxygen partial pressure (*p*O_2_) is measured in the channel fluid using Ruthenium micelles fluorescence (see Materials and Methods). (A) *p*O_2_ in empty channels at different flow rates (0; 1 and 2 ml/h), in 250 μm- (red) and 1 mm- (blue) height channels. (B) *p*O_2_ in 250 μm- height channel at 1 ml/h, in the presence (dark red) or absence (light red) of a growing biofilm 12 hours after initiation. (C) *p*O_2_ vs. flow rate in a 250 μm—height channel in the presence of the growing biofilm. Error bars (smaller than dots in C) represent standard deviation over two measurements.

We first measured O_2_ content in the 1 mm- and 250 μm- height channels supplied with growth medium at 1 mL/h in the absence of biofilm to evaluate the possible effect of channel geometry on oxygen availability. We found no significant difference between the two channels—the partial pressures were found equal to 5.0±0.1% and 5.3±0.2% for the 1 mm and 250 μm-height channels, respectively. Increasing the flow rate up to 2 mL/h did not modify the O_2_ levels (Fig 8A). These values, which were also found in the M63B1-glucose stock solution, are typical of resting aqueous solutions in contact with atmospheric air. Besides, when flow was stopped, we measured slightly higher levels of O_2_, suggesting that the PDMS walls, which are intrinsically permeable to O_2_, released their O_2_ content in the channel lumen in the absence of flow—an O_2_ content higher in PDMS than in the surrounding air has already been shown elsewhere[50].

Next, we monitored oxygen level in the 250 μm-height channel in the presence of a biofilm after 12 hours of growth. Only the advancing-front growth mode, which preserved a central region devoid of biofilm in the channel, allowed probe fluorescence intensity interpretation in terms of O_2_ concentration in the flow. Thereby, we found a stationary O_2_ level significantly reduced in comparison with the empty channels: the *p*O_2_ value was 1.2±0.1%, vs. 5% in the empty channels. This drop accounted for O_2_ consumption by the biofilm and confirmed the low-oxygen stress experienced by the biofilm grown under these conditions (Fig 8B). O_2_ thus appears as a potentially limited resource under confined flow conditions.

Increasing the flow rate by a factor of 4 in the 250 μm-height channel induced an increase of the channel fluid oxygen content (Fig 8C). The non-monotonical shape of the curve may result from a reduction of the net balance of O_2_ under conditions where the influx of advected O_2_ does not compensate the removal by the flow of the O_2_ contributed to the fluid by diffusion from the PDMS. The results displayed in Fig 8C indicates that advection rate contributes to O_2_ equilibrium in such millifluidic channels. However, it's worth noting that we measured oxygen content in the channel fluid flow, which is not necessarily unequivocally related to the O_2_ level sensed by the cells in the biofilm. Besides, while the different growth rates measured in the biofilms growing in our 250 μm- and 1 mm-height channels might arise from different oxygen levels, the distinct development patterns exhibited by these biofilms may also directly impact the growth rate.

The main result we obtained from this evaluation of environmental O_2_ concentration is that biofilms under flow in millifluidic channels grow under low oxygen level. This is consistent with the modulation of the micro-aerobiosis functions revealed by the transcriptomics data.

## Discussion

We have disentangled the features that impact bacterial biofilm formation under hydrodynamic stress, a ubiquitous process in nature (including living hosts) and in industrial devices. To date, the question of flow effects on biofilm development has remained confused, very likely because fluid flow simultaneously determines the amplitude of several driving forces very distinct by nature but technically difficult to separate, such as mechanical stress and solute transport.

Here, we tackled this problem by using millifluidic channels of variable height to apply a range of shear stresses under the same flow rate and a limited variation of the advection rate.

Based on this setup, we first recognized, using the theoretical velocity expressions of O’Brien[40], that millifluidic geometries provided shear stress values already broadly distributed within a unique channel. While a growing number of investigations of biofilms under flow are using flow cell channels with similar dimensions[5, 21, 51, 52], this property has essentially been overlooked so far.

Our strategy consisted in achieving real-time monitoring of the biofilm growth to quantitatively assess its spatiotemporal development under controlled shear stress conditions. This approach enabled to disentangle the settling initiation events and the subsequent growth of the material under flow.

Looking into the details of the initial biofilm development patterns and kinetics within the five channels of our setup, we found a mechanical control of biofilm settlement. Above a threshold shear stress value of 10 mPa, no direct initiation of the biofilm on the surface could occur. Interestingly, if we evaluate the tangential force, *F* = σ. *A*, applied by such a shear stress on a surface *A* of 1 μm^2^ (approx. the average projected surface of an *E*. *coli*), we obtain a value of 10^−2^ pN. Using a more sophisticated evaluation of the drag force, as in De La Fuente et al.[53], also yields a value below 0.1 pN. Such forces acting over 1 μm will barely engage a few *k*_*B*_*T*, which is lower than the energy required to break molecular bonds attaching a bacterium to a surface. Forces needed to break even a single bond would be of the order of a few to a few tens of piconewtons[54, 55]. Our finding thus suggests that only weak interaction forces such as hydration, van der Waals or electrostatic forces compatible with an amplitude of some hundredths of piconewtons[56, 57] retain bacteria on the surface during the initiation phase. This soft adhesion is sufficient to enable cells to resist shear stress up to a 10 mPa threshold and to start building a biofilm. Such weak forces have rarely been reported to interfere with biofilm formation, very likely due to the authors’ usual focus on forces required to remove mature biofilm, which appear to be different from forces hindering initial adhesion[57]. Worthy of mention is the fact that the exact amount of energy engaged in this surface initial dwelling of bacteria will undoubtedly depend on the precise physicochemical properties of the cell and of the substrate surfaces but it is important to notice that even weak cell/surface interactions may eventually lead to a mature robust biofilm. Given the increase of the apparent lag time of biofilm growth with shear stress, we also suggest that flow might delay the formation of this conditioning layer, simply by partially washing out the molecules secreted by the cells. This hypothesis, which has not received much attention so far, would deserve further investigation to decide whether a hydrodynamic, mechanical forces-independent mechanism simply driven by diffusion and advection, participates in the control of biofilm initiation.

Next, we addressed the question of the biological response of biofilms to shear stress as a mechanical force, focusing on biofilm growth. Through initiation control, hydrodynamics oriented biofilm growth towards two different modes: one under low shear stress, in which biofilm grows from uniform surface attachment—that we called uniform growth mode—and one under higher shear stress in which biofilm grows from the channel side walls after a delay—the advancing-front growth mode.

We demonstrated that the biofilms growing in the uniform mode at bottom shear stress values ranging from 0.42 to 10 mPa (32 growth curves) all displayed the same growth rate, indicating that the intensity of mechanical stress did not impact biofilm growth rate *per se*. In good agreement with this finding, the global transcriptome analysis that we performed on biofilms grown at low and high shear stress exhibited no significant differential expression of mechanosensing-related genes, such as *flh* or *fli* genes related to flagellar function[58, 59], *msc* genes coding for membrane stress-sensing ionic channels[35], or even *csg*, *cpx* or *rpoS* genes involved in more generic surface-sensing systems[60].

The absence of significant mechano-specific biological signaling in the biofilm, in response to the environmental mechanical stress increase, is consistent with the fact that no fluid flow penetrated into the biofilm. Indeed, the latter observation implies that most of the biofilm-dwelling cells live protected from mechanical stress, which is thus expected to only affect the interface layer. At this point, it is important to discuss the limitations of biofilm transcriptional profiling. In particular, the whole transcriptome captures a gene expression pattern averaged over the whole cell population, regardless of the strong spatial heterogeneity of the biofilm and of the conditions[61]. Thus, a small sub-population with specific expression levels would be missed by this analysis, and we cannot exclude overlooking the response of a minority population such as an interface layer, for instance. Besides, the differential transcriptomic analysis principally pointed to a set of genes involved in cell micro-aerobiosis, indicating that the biofilm grown under lower fluid dynamics exhibited enhanced low-O_2_ stress than its higher fluid dynamics counterpart. In addition, four genes involved in cell resistance to acidic conditions are more strongly expressed at lower fluid dynamics—possibly regarding metabolite removal by the flow—and 3 genes related to sulfate metabolism, were less expressed. The micro-aerobiosis genes also appeared to be more strongly expressed in cells from both channels than in planktonic agitated log-phase cells, as expected. The micro-aerobiosis genes have only been previously mentioned in transcriptomic characterization of biofilm traits by Schembri et al.[48, 62], very likely due to the extreme sensitivity of transcriptomes to experimental conditions. As pointed out by Beloin and Ghigo[62], transcriptomes obtained on *E*. *coli* biofilm-dwelling cells are difficult to compare across laboratories. In addition, very few transcriptomic analyses have been performed on biofilms under flow. Yet, looking at the top 20 genes sorted in our analysis when comparing the gene expression in the biofilm under low fluid dynamics with the one in an exponentially growing cell culture, we found that in our analysis, 15 genes are in common with the list published by Schembri and collaborators[48, 63] (S3 Table) whose conditions are close to ours. By contrast, only one gene was found in common with the list of Beloin and collaborators[63] whose setup consisted in a slide immersed in a quasi-static chemostat for 8 days. Nevertheless, despite the low level of citations of the low O_2_- stress genes in the whole transcriptome studies dedicated to biofilms, more and more studies demonstrate the role of O_2_ availability as a pivotal driving force underlying bacterial community behavior[64–66].

Our results indicated that biofilms under flow in milllifluidic channels experience low-O_2_ medium conditions consistent with the micro-aerobiosis detected by the transcriptomic analysis. The reduced low-O_2_ stress observed under the higher fluid dynamics conditions indicates that in the advancing-front mode, the biofilm had a better access to O_2_. This may be due to its distinct pattern, advantageous to benefit from the different sources of O_2_ in the millifluidic channel, such as the environmental air transported through the PDMS walls. However, it may also be due to the slightly higher advection rate. Further elucidation of the causal relationships linking hydrodynamics, oxygen level and biofilm growth would require additional experiments enabling *in situ* determination of O_2_ distribution within the biofilm under flow.

Our findings also highlight a stress-escaping strategy developed by this *E*. *coli* strain to finally settle biofilm in areas initially precluded due to too high shear stress, such as the center of the strongly confined channels. This strategy consists in settling in flow-protected borders before quickly expanding—albeit after a delay—towards the previously forbidden zones, in a more mature and topologically favorable form. This is a crucial result to have in mind to conceive fluidic circuits where bacteria will pass and try to dwell. Should you aim at fighting against or at domesticating the biofilm, the flow properties at the device boundaries should be carefully examined.

## Conclusions

Hydrodynamics has an exquisite influence on the development of 3D biofilm. Under laminar flow and mild shear stress, mechanical forces have a veto power on initial surface colonization. It induces a spatial patterning of the biofilm that reflects settlement in zones of permissive shear stress. However, no mechanotransduction process has been found here to significantly contribute to biofilm physiological properties. Biofilm appears as a bacterial strategic bypass of the hydrodynamic barrier opposed to their surface invasion: settling in shear stress protected areas, they dwell there long enough to mature into a material able to expand in the previously forbidden areas, although with a delay and a modified pattern. Hydrodynamic molecular transport essentially impacts the subsequent growth phase of the biofilm. The control of oxygen distribution—a key factor of the biofilm lifestyle—appears as an essential regulation element carried by the fluid flow.

These features are of utmost importance to understand and anticipate biofilm development as well as to achieve informed design of fluidic setups either to investigate biofilm fundamental properties or to engineer and control these adherent bacterial communities.

## Supporting information

S1 File Estimation of glucose concentration. (PDF)

S1 Video Biofilm growth imaging over 20 hours in 1 mm-height channel. Biofilm initiated as in Material and Methods section. Movie starts at the same time as image acquisition. Brightfield using a 20X objective. Acquisition time is 20msec, one frame every 5 mins. All other details as in Fig 2A.
(AVI)

S2 Video Biofilm growth imaging over 20 hours in 750 μm-height channel. Biofilm initiated as in Material and Methods section. Movie starts at the same time as image acquisition. Brightfield using a 20X objective. Acquisition time is 20msec, one frame every 5 mins. All other details as in Fig 2A.
(AVI)

S3 Video Biofilm growth imaging over 20 hours in 500 μm-height channel. Biofilm initiated as in Material and Methods section. Movie starts at the same time as image acquisition. Brightfield using a 20X objective. Acquisition time is 20msec, one frame every 5 mins. All other details as in Fig 2A.
(AVI)

S4 Video Biofilm growth imaging over 20 hours in 350 μm-height channel. Biofilm initiated as in Material and Methods section. Movie starts at the same time as image acquisition. Brightfield using a 20X objective. Acquisition time is 20msec, one frame every 5 mins. All other details as in Fig 2A.
(AVI)

S5 Video Biofilm growth imaging over 20 hours in 250 μm-height channel. Biofilm initiated as in Material and Methods section. Movie starts at the same time as image acquisition. Brightfield using a 20X objective. Acquisition time is 20msec, one frame every 5 mins. All other details as in Fig 2A.
(AVI)

S1 Fig Signal calibration. (A) Microphotometry measurements. The natural logarithm of the light attenuation factor—ln(*I*_*0*_/*I*), so-called microscopic absorbance *A*_μ_—was derived from microscope image intensities obtained on methylene blue and biofilm samples of different concentrations in the millifluidic channel series. Then *A*_μ_ was plotted against sample concentrations determined independently.
For methylen blue (MBlue) (blue triangles), we used solutions of known concentrations and considered the quantity [MBlue].*h*, i.e. the mass concentration multiplied by the height of the measured channel, to take into account the path of the light in the solution (top *x*-axis).
For biofilms (open circles) we extracted the material from the channels using repeated air jet pulses and thoroughly dispersed it in 300 μl of minimum medium to measure optical density at 600 nm (macroscopic OD). Considering an OD-specific concentration of *E*. *coli* cells in a suspension, i.e. the number of cells per milliliter at an OD of 1 measured at 600 nm, equal to 5.10^8^ cells/mL, we deduced the number of cells per channel (bottom x-axis).
The analogue absorbance, ln(*I*_*0*_/*I*), derived from microscope images, increased mostly linearly with sample mass concentration up to approx. 60% attenuation (ln(*I*_*0*_/*I*) = 0.35). Interestingly, the same behavior regarding linearity deviation was observed for MBlue solutions and biofilms, indicating that light attenuation measured in microscopy on biofilms approx. 100 μm in height was not significantly impaired by scattering. Therefore, the analogue absorbance could be taken as an accurate mass concentration proxy, provided that the proportionality factor and the limit of the linear regime can be determined.
**(B)** Incident light (*I*_*0*_) effect on analogue absorbance-mass concentration relationship. A ten-channel series was prepared with increasing concentrations of methylene blue and the analogue absorbance was measured for two different intensities of incident light; (black circles) *I*_*0*_ = 2140 ±110 a.u. and (gray triangles) *I*_*0*_ = 526±20 a.u. Increasing incident light intensity increases the range of quasi-linearity of the analogue absorbance-concentration relation. Quantitative analyses of biofilm growth experiments were performed using *I*_*0*_ = 2000±100 a.u. ensuring a quasi-linear detection of the biomass in the channel up to an absorbance value of 0.7; i.e. an attenuation of 50%.
From these data, we obtained the relationship linking the absorbance *A*_μ_ and the number *N*_*ch*_ of cells per channel: *A*_μ_ = (1.1±0.2)x10^-9^ *N*_*ch*_. Then, assuming uniform colonization of the channel, we deduced the relationship linking A_μ_ and the surfacic concentration *C*_*s*_, defined as the number of cells dwelling over 1 mm^2^ channel surface, i.e. *C*_*s*_ = (3.0±0.2).x10^7^ *A*_μ_, by dividing *N*_*ch*_ by the channel surface 30 mm^2^.
In our quantitative analysis of biofilm growth curves (see main text and Figs 3–5), we took into account only data with measured absorbance values <0.74, in order to remain in the quasi-linear region of the absorbance-concentration (or absorbance-biomass) relation. To further correct for the slight nonlinearities present even at low absorbance values in this relation, we used the experimentally-measured relation in panel (B), yielding the equivalent [Mblue].h corresponding to each measured absorbance. This equivalent [Mblue].h is termed “corrected absorbance” in the main text. This correction, which can be visualized on Fig 3, yields better exponential adjustments.
(TIF)

S2 Fig Velocity field in channels without biofilm. **A**: Velocity field in a channel of height *h* = 1 mm without biofilm. Dots: experimentally-measured velocities *v* at various positions *(y*,*z)* across the channel. Surface: adjustment to the theoretical expression of the velocity field of a viscous fluid in a rectangular channel (Eq 2). The color scale denotes velocities (see *v* axis). Black lines: distance between the experimentally-measured velocities and the adjusted values at the same location. Both the height *h* and the volumetric flow rate *Q* (through—η *dp/dx*) were adjusted, as in the presence of the biofilm (Fig 6), yielding *h* = 1.00 mm as expected, and *Q* = 0.83 mL/h (nominal value 1 mL/h). The coefficient of determination of the fit is R^2^ = 0.92. Since the velocity flow in this biofilm-free channel was consistent with a flow rate slightly lower than the nominal value, the flow rate *Q* was also adjusted in the same channel with growing biofilm (Fig 6A). In that case, since the flow rate is kept constant during the experiment, we adjusted the velocity field separately at each time point (with *h* as the only adjustable parameter) at given imposed values of *Q*, and we then chose the one that minimized the total sum of the squared residues over all times, yielding *Q* = 0.85 mL/h, consistent with the adjusted value *Q* = 0.83 mL/h obtained here.
**B**: Velocity field in a channel of height *h* = 250 μm without biofilm. Dots: experimentally-measured velocities *v* at various positions *(y*, *z)* across the channel. Surface: adjustment to the theoretical expression of the velocity field of a viscous fluid in a rectangular channel (Eq 2) The color scale denotes velocities (see *v* axis) and is the same as in Fig S2A for the sake of comparison. Black lines: distance between the experimentally-measured velocities and the adjusted values at the same location. Both the width *w* and the effective position *y*_0_ of the no-slip boundary condition in the *y* direction were adjusted, as in the presence of the biofilm (Fig 1), yielding *w* = 1.08 mm and *y*_0_ = 6.5 μm, consistent with the expectations (*w* = 1.0 mm and *y*_0_ = 0). The volumetric flow rate *Q* was set to its experimentally-imposed nominal value 1 mL/h. (Note that choosing instead to adjust *Q* gives consistent results, e.g. *Q* = 0.97 mL/h in the presence of the biofilm.) The coefficient of determination of the fit is R^2^ = 0.97.
(TIF)

S3 Fig Experimental velocity measurement. Picture of red fluorescent particles (exc 580 nm/ em 605 nm) 1 μm in diameter flowing in a 1 mm-height channel under continuous medium supply at a nominal flow rate of 1 ml/h. Image recorded with a 40 msec acquisition time. z-stack images are collected using a *z* spacing of 5 μm. The displayed image has been recorded at z = 900 μm. Only on-focus trails, as shown in the insert, are taken into account for the velocity field determination.
(TIF)

S4 Fig Differential expression of the genes coding for cytochrome bd and bo oxidases. Consistently with the hypothesis that bacteria in L samples experienced lower level of O_2_ than in H samples, we found that the genes coding for cytochrome bd and bo oxidases, the activity of which is known to be related to the fraction of aerobiosis (Alexeeva, S., Hellingwerf, K. J. & Teixeira de Mattos, M. J. Quantitative assessment of oxygen availability: perceived aerobiosis and its effect on flux distribution in the respiratory chain of Escherichia coli. *J Bacteriol* **184**, 1402–1406 (2002)), were differently expressed in H and L (although with an FDR>0.01); the levels of expression in samples E are also consistent with a better level of oxygenation in samples E, as expected. Left panel: expression of *cbdAB* coding for cytochrome bd oxidase, which contributes to respiratory activity below 50% aerobiosis; expression is higher in samples L compared to samples H, and genes are repressed in samples E. Right panel: expression of *cyoABCDE* coding for cytochrome bo oxidase, which contributes significantly to respiratory activity above 55% aerobiosis; expression is higher in samples E and lower in samples L, consistent with a better oxygenation in samples E and a lower oxygenation in samples L. Bars: SDs.
(TIF)

S5 Fig Kymographs of biofilm development under different hydrodynamical conditions. On each kymograph, the length scales on the top axis stand for the distance from the edge of the channel while the time scale on the right reports time elapsed from the start of the flow. (a) 0.5 mm-height channel under 1 mL/h flow rate, i.e. shear stress up to 9 mPa; the uniform growth mode is observed, indicating that the colonization shear stress threshold is above 9 mPa. (b) 0.35 and (c) 0.25 mm height channels under 1 mL/h flow rate; the limit for the initial colonization corresponds in these channels to shear stress values of 10 mPa and 13mPa, respectively. (d) A 0.5 mm-height channel was run at a different flow rate of 2mL/h; the limit of initial colonization consistent with a shear stress colonization threshold of 11 mPa. Dashed lines indicate initial shear stress whitin the fcolonization.
(TIF)

S1 Table Data statistical dispersion. Means and standard deviations over at least three distinct channels.
(PDF)

S2 Table Growth rates are higher in the higher fluid dynamical regime. Growth rate values derived from the exponential fit of the biomass growth kinetics in the different channels (μ).
(PDF)

S3 Table The 20 genes most affected in expression: biofilm versus exponential growth phase; comparison with two other experiments. (PDF)
