# Supplementary material for: Bacterial biofilm under flow: First a physical struggle to stay, then a matter of breathing
Source: PLoS One. 2017 Apr 12;12(4):e0175197. doi: 10.1371/journal.pone.0175197 (PMC5389662; doi:10.1371/journal.pone.0175197)
Supplement: S1 File — (PDF) [file pone.0175197.s001.pdf]

Estimation of glucose concentration

Let us consider a channel of height h, width w and length L. We first consider the case of a biofilm attached to the channel bottom surface at z=0 with a thickness e<<h, under continuous supply of a medium, and that consumes glucose.
A molecule of glucose entering the channel at height $z=z_0$ takes a mean time $t_{ADV}=x_0/\langle v\rangle$ to reach the distance x_0, where $\langle v\rangle$ is the mean fluid velocity. The mean time taken to diffuse to the channel bottom at z=0 from a height z_0 is $t_{DIF}= z_0^2/D$, where D is the diffusion coefficient of glucose. These two times define a curve:

$$z^*(x)=(x\, D/\langle v\rangle)^{1/2} \qquad (1)$$

below which the concentration of glucose is less than c_0 because of the consumption by the biofilm, and above which the concentration of glucose is c_0. Here c_0 is the concentration of glucose in the culture medium.
The flow of glucose at $z=e\approx 0$ through a horizontal slice of area A is equal to C, the number of molecules of glucose consumed per second by the bacterial biofilm within the slice.
To estimate the concentration c_S of glucose just above the slice, let the current of glucose along the z axis be:

$$j_Z(x)=-D\,(c_0-c_S)/z^*(x)= -C/A \qquad (2)$$

By combining (1), (2) and $Q=\langle v\rangle\, w\, h$, where Q is the fluid flow rate, c_S can be estimated by:

$$c_S(x)= c_0 - C/A *(x\, h\, w/(Q\, D))^{1/2} \qquad (3)$$

with c_0=1.36x10^{24} molecules/m^3 (corresponding to 0.4% glucose), D=5.7x10^{-10} m^2/s and Q=1 ml/h =2.78x10^{-10} m^3/s.
C/A is the consumption of glucose by unit area. The experimental data (recovering biofilm cells and measuring the optical density, not shown) suggest that there are ≈10^9 bacteria in a biofilm. By taking a maximum consumption rate c_M=197 molecules of glucose per second per cell (Natarajan, A. & Srienc, F. Dynamics of glucose uptake by single Escherichia coli cells. *Metab Eng* **1**, 320-333, (1999)), we estimate: $C/A= c_M/(hw)$=6.6x10^{15} molecules of glucose per m^2 per s.
The lowest concentration of glucose is found at the bottom of the channel near the outlet, at $(x,y,z)=(L,y,0)$, and is:

$$c_S(L)= c_0-C/A *(L\, h\, w/Q\, D)^{1/2} \qquad (4)$$

It allows us to estimate c_S/c_0 to be 0.998 in the 1 mm height channel, and 0.999 in the 250 µm height channel.
If we take into account the variation of velocity along the z axis, using a Poiseuille velocity field, it does not affect significantly the result (not shown).
In the 250 µm height channel, the biofilm grows from the edges (see text). The estimation of c_S in that case can be obtained by considering two slices of biofilm growing at z=0 and $z=h$, in a channel where (w,h,L)=(0.25 mm, 1 mm, 30 mm). The function $z^*(x)$ is the same as (1) and gives $z^*(L)$ =0.124 mm < $h/2$; thus, the glucose-depleted regions generated by both biofilm slices, where $c<c_0$, do not overlap. The lowest concentration of glucose is then calculated at $x=L$ using (3), which still gives (4).
In this estimate, the y-dependence of velocity has been neglected.

Experimental clues

The above theoretical evaluation was confirmed by experimental observations showing that no significant biofilm growth decrease occurred along the channel *x* axis (direction of the flow) — if the glucose were depleted, the biofilm would be progressively less dense toward the channel outlet. We measured light transmission *I* along the channel length *x* from the inlet to the outlet, for two 1 mm-height channels, in the presence of biofilm after 24h growth; Figure SI1 shows $\ln(I_0/I)$ vs *x*.

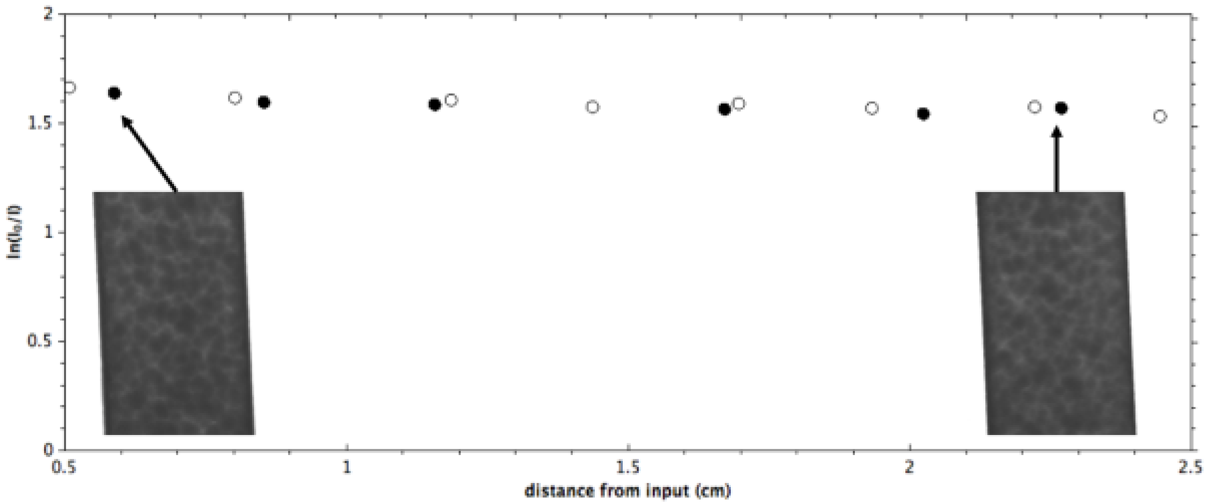

Figure SI1: $\ln(I_0/I)$ vs *x*, distance from channel inlet in cm (the length of a channel is 3 cm; *x*=0 corresponds to the channel inlet). Filled and open circles: measurements in two different 1 mm height channels after 24 h of biofilm growth. Pictures: snapshots — field of view: 1 mm x 1.65 mm — of one channel taken with a 4x objective, near inlet (left) and near outlet (right), as indicated by the arrows; no significant differences are observed in biofilm microscopic absorbance.
