# Supplementary figures and images for: Bacterial biofilm under flow: First a physical struggle to stay, then a matter of breathing
Source: PLoS One. 2017 Apr 12;12(4):e0175197. doi: 10.1371/journal.pone.0175197 (PMC5389662; doi:10.1371/journal.pone.0175197)

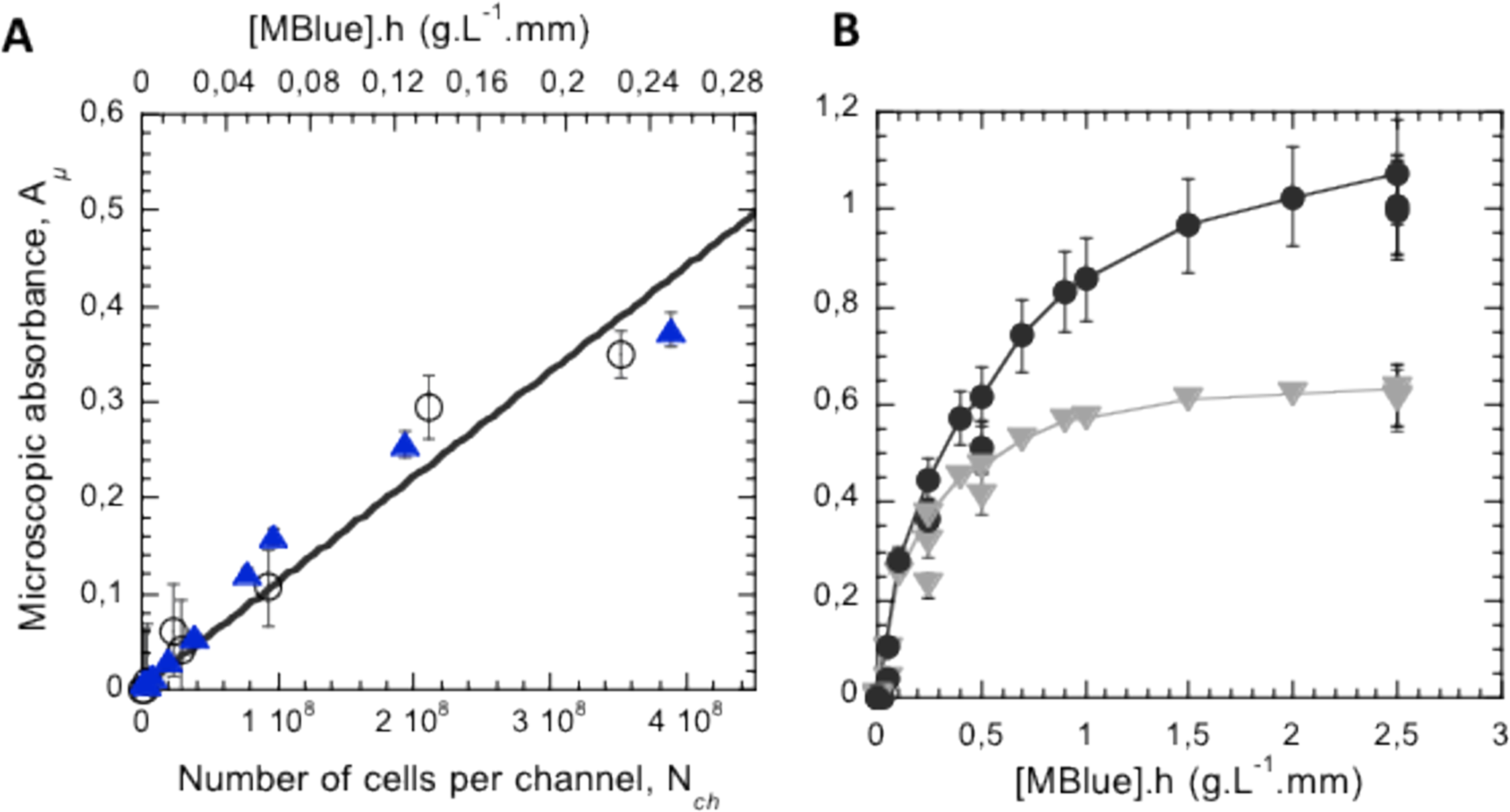

Supplement: S1 Fig — (A) Microphotometry measurements. The natural logarithm of the light attenuation factor—ln(I0/I), so-called microscopic absorbance Aμ—was derived from microscope image intensities obtained on methylene blue and biofilm samples of different concentrations in the millifluidic channel series. Then Aμ was plotted against sample concentrations determined independently. For methylen blue (MBlue) (blue triangles), we used solutions of known concentrations and considered the quantity [MBlue].h, i.e. the mass concentration multiplied by the height of the measured channel, to take into account the path of the light in the solution (top x-axis). For biofilms (open circles) we extracted the material from the channels using repeated air jet pulses and thoroughly dispersed it in 300 μl of minimum medium to measure optical density at 600 nm (macroscopic OD). Considering an OD-specific concentration of E. coli cells in a suspension, i.e. the number of cells per milliliter at an OD of 1 measured at 600 nm, equal to 5.108 cells/mL, we deduced the number of cells per channel (bottom x-axis). The analogue absorbance, ln(I0/I), derived from microscope images, increased mostly linearly with sample mass concentration up to approx. 60% attenuation (ln(I0/I) = 0.35). Interestingly, the same behavior regarding linearity deviation was observed for MBlue solutions and biofilms, indicating that light attenuation measured in microscopy on biofilms approx. 100 μm in height was not significantly impaired by scattering. Therefore, the analogue absorbance could be taken as an accurate mass concentration proxy, provided that the proportionality factor and the limit of the linear regime can be determined. (B) Incident light (I0) effect on analogue absorbance-mass concentration relationship. A ten-channel series was prepared with increasing concentrations of methylene blue and the analogue absorbance was measured for two different intensities of incident light; (black circles) I0 = 2140 ±110 a.u. and ( [file pone.0175197.s007.tif]

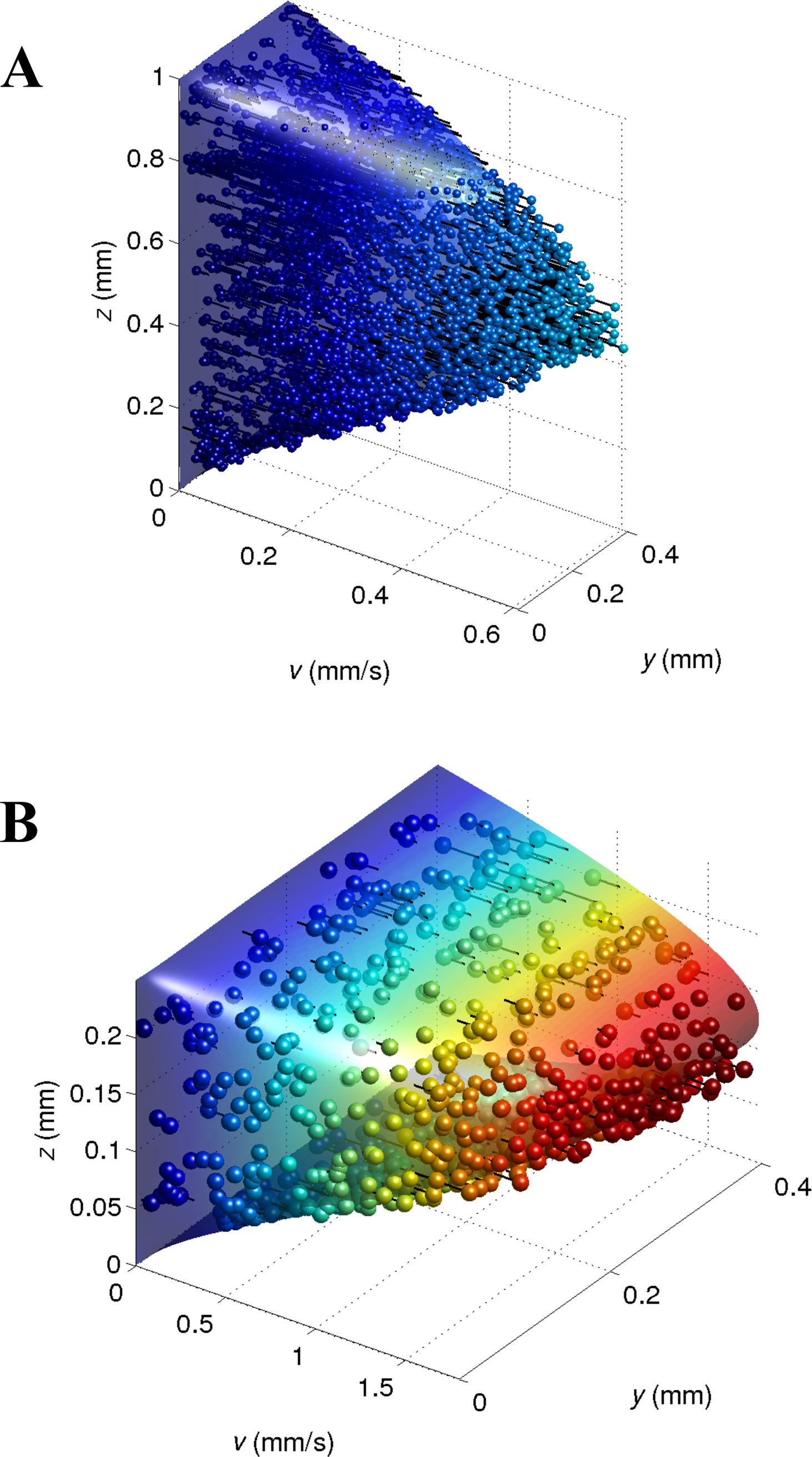

Supplement: S2 Fig — A: Velocity field in a channel of height h = 1 mm without biofilm. Dots: experimentally-measured velocities v at various positions (y,z) across the channel. Surface: adjustment to the theoretical expression of the velocity field of a viscous fluid in a rectangular channel (Eq 2). The color scale denotes velocities (see v axis). Black lines: distance between the experimentally-measured velocities and the adjusted values at the same location. Both the height h and the volumetric flow rate Q (through—η dp/dx) were adjusted, as in the presence of the biofilm (Fig 6), yielding h = 1.00 mm as expected, and Q = 0.83 mL/h (nominal value 1 mL/h). The coefficient of determination of the fit is R2 = 0.92. Since the velocity flow in this biofilm-free channel was consistent with a flow rate slightly lower than the nominal value, the flow rate Q was also adjusted in the same channel with growing biofilm (Fig 6A). In that case, since the flow rate is kept constant during the experiment, we adjusted the velocity field separately at each time point (with h as the only adjustable parameter) at given imposed values of Q, and we then chose the one that minimized the total sum of the squared residues over all times, yielding Q = 0.85 mL/h, consistent with the adjusted value Q = 0.83 mL/h obtained here. B: Velocity field in a channel of height h = 250 μm without biofilm. Dots: experimentally-measured velocities v at various positions (y, z) across the channel. Surface: adjustment to the theoretical expression of the velocity field of a viscous fluid in a rectangular channel (Eq 2) The color scale denotes velocities (see v axis) and is the same as in Fig S2A for the sake of comparison. Black lines: distance between the experimentally-measured velocities and the adjusted values at the same location. Both the width w and the effective position y0 of the no-slip boundary condition in the y direction were adjusted, as in the presence of the biofilm (Fig 1), yielding w = 1.08 mm and y0 = 6.5 μ [file pone.0175197.s008.tif]

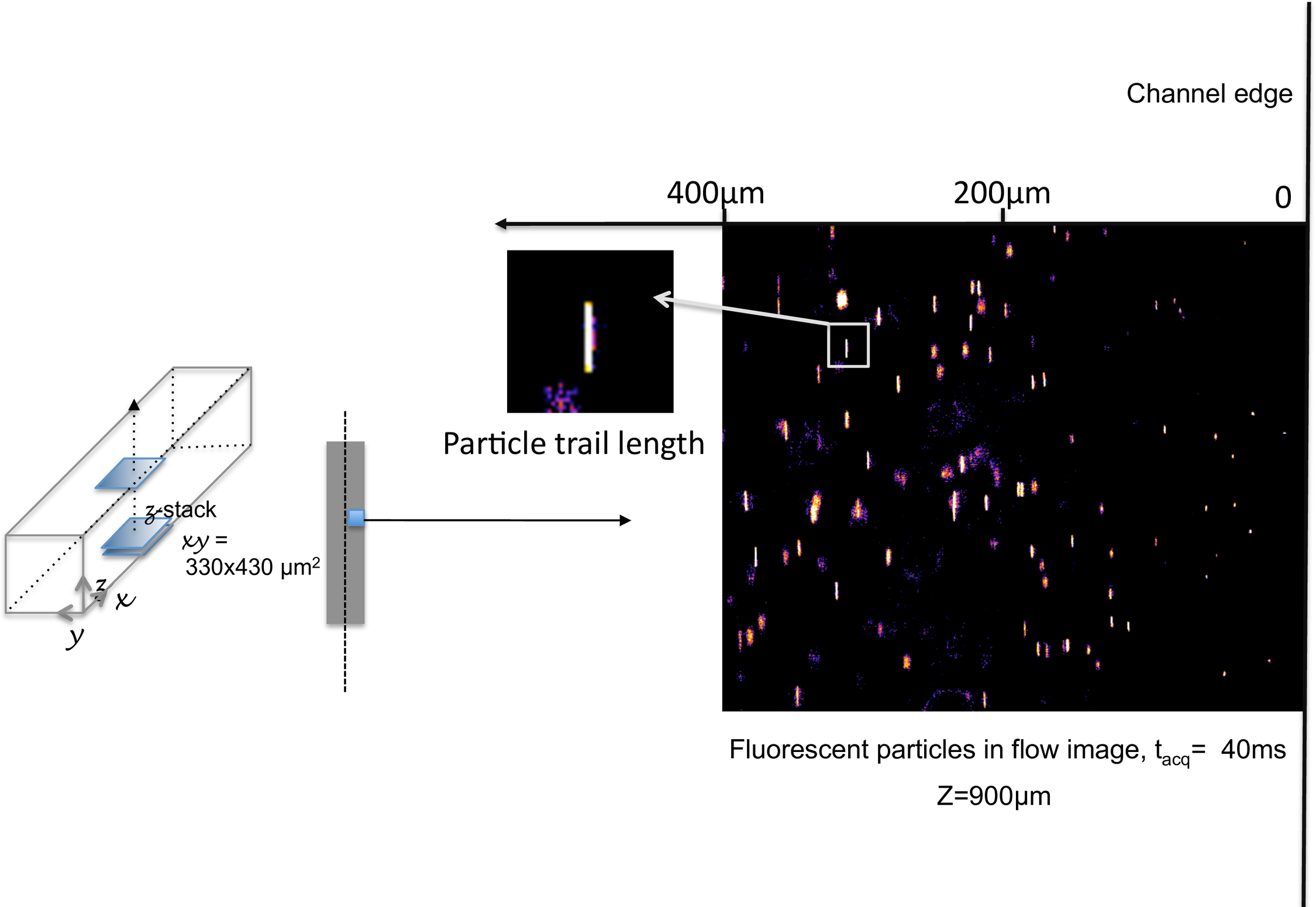

Supplement: S3 Fig — Picture of red fluorescent particles (exc 580 nm/ em 605 nm) 1 μm in diameter flowing in a 1 mm-height channel under continuous medium supply at a nominal flow rate of 1 ml/h. Image recorded with a 40 msec acquisition time. z-stack images are collected using a z spacing of 5 μm. The displayed image has been recorded at z = 900 μm. Only on-focus trails, as shown in the insert, are taken into account for the velocity field determination. (TIF) [file pone.0175197.s009.tif]

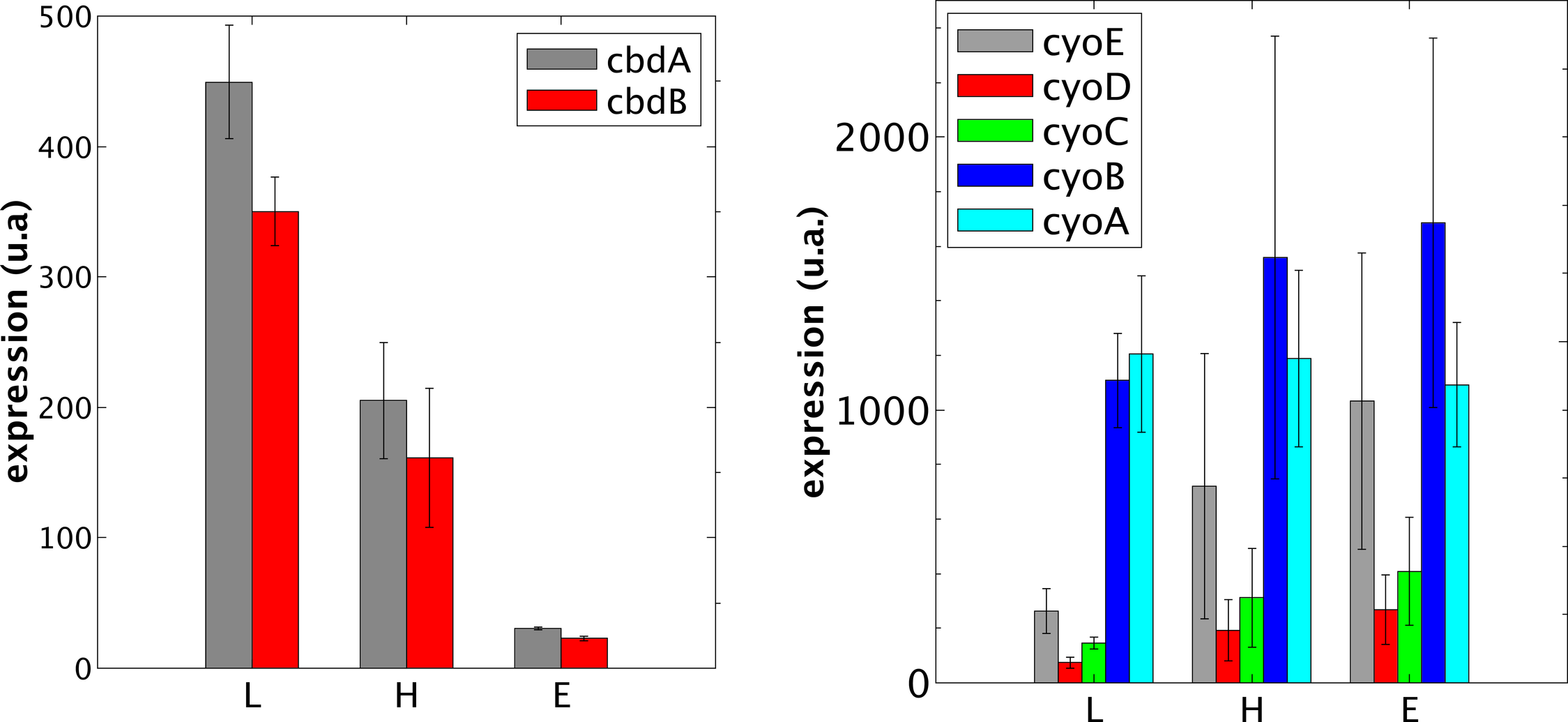

Supplement: S4 Fig — Consistently with the hypothesis that bacteria in L samples experienced lower level of O2 than in H samples, we found that the genes coding for cytochrome bd and bo oxidases, the activity of which is known to be related to the fraction of aerobiosis (Alexeeva, S., Hellingwerf, K. J. & Teixeira de Mattos, M. J. Quantitative assessment of oxygen availability: perceived aerobiosis and its effect on flux distribution in the respiratory chain of Escherichia coli. J Bacteriol 184, 1402–1406 (2002)), were differently expressed in H and L (although with an FDR>0.01); the levels of expression in samples E are also consistent with a better level of oxygenation in samples E, as expected. Left panel: expression of cbdAB coding for cytochrome bd oxidase, which contributes to respiratory activity below 50% aerobiosis; expression is higher in samples L compared to samples H, and genes are repressed in samples E. Right panel: expression of cyoABCDE coding for cytochrome bo oxidase, which contributes significantly to respiratory activity above 55% aerobiosis; expression is higher in samples E and lower in samples L, consistent with a better oxygenation in samples E and a lower oxygenation in samples L. Bars: SDs. (TIF) [file pone.0175197.s010.tif]

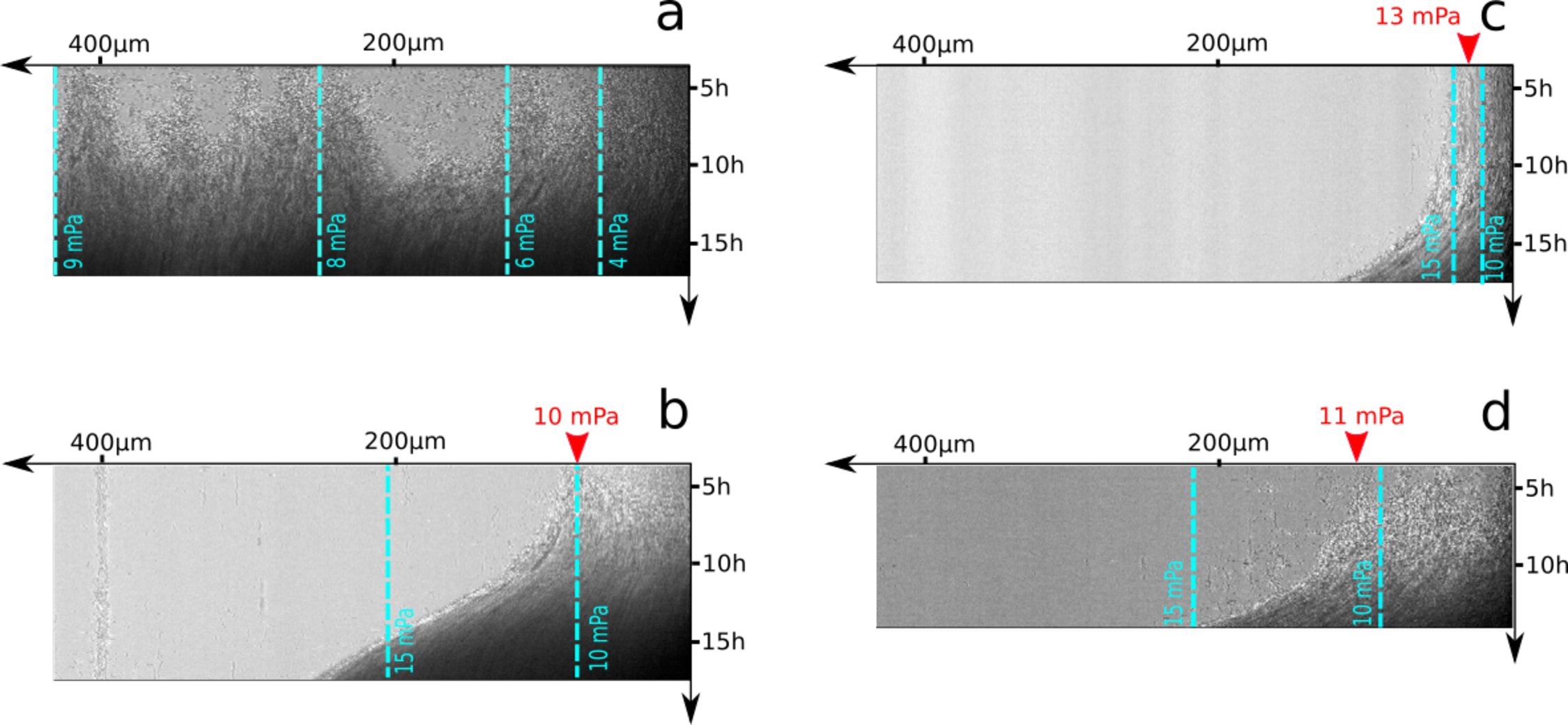

Supplement: S5 Fig — On each kymograph, the length scales on the top axis stand for the distance from the edge of the channel while the time scale on the right reports time elapsed from the start of the flow. (a) 0.5 mm-height channel under 1 mL/h flow rate, i.e. shear stress up to 9 mPa; the uniform growth mode is observed, indicating that the colonization shear stress threshold is above 9 mPa. (b) 0.35 and (c) 0.25 mm height channels under 1 mL/h flow rate; the limit for the initial colonization corresponds in these channels to shear stress values of 10 mPa and 13mPa, respectively. (d) A 0.5 mm-height channel was run at a different flow rate of 2mL/h; the limit of initial colonization consistent with a shear stress colonization threshold of 11 mPa. Dashed lines indicate initial shear stress whitin the fcolonization. (TIF) [file pone.0175197.s011.tif]
