# Supplementary material for: Bacterial biofilm under flow: First a physical struggle to stay, then a matter of breathing
Source: PLoS One. 2017 Apr 12;12(4):e0175197. doi: 10.1371/journal.pone.0175197 (PMC5389662; doi:10.1371/journal.pone.0175197)
Supplement: S1 Table — Means and standard deviations over at least three distinct channels. (PDF) [file pone.0175197.s012.pdf]

S1 Table.

250 μm-height channel						
ROI#		Mean μ (d^{-1})	SD(μ)		Mean δ	SD (δ)
1		12.0	1.6		0.42	0.07
2		20.6	4.3		0.54	0.04
3		26.6	5.6		0.61	0.10
4		28.9	11.0		0.69	0.11
5		24.0	7.0		0.75	0.12
1mm-height channel						
1		13.4	2.4		0.33	0.02
2		12.4	2.2		0.36	0.01
3		11.7	0.5		0.36	0.05
4		12.4	1.1		0.35	0.03
5		13.8	2.6		0.36	0.03
6		13.1	1.9		0.38	0.02
7		13.8	2.3		0.39	0.04
8		13.6	0.1		0.42	0.08
9		14.3*	0.4*		0.39 *	0.03*
10		14.2*	0.4*		0.46*	0.01*

∗Only 2 experiments
