# Supplementary material for: Bacterial biofilm under flow: First a physical struggle to stay, then a matter of breathing
Source: PLoS One. 2017 Apr 12;12(4):e0175197. doi: 10.1371/journal.pone.0175197 (PMC5389662; doi:10.1371/journal.pone.0175197)
Supplement: S2 Table — Growth rate values derived from the exponential fit of the biomass growth kinetics in the different channels (μ). (PDF) [file pone.0175197.s013.pdf]

S2 Table.

Channel height (mm)	Max μ*(d^{-1})
1	14.3
0.75	15.7
0.5	14.7
0.35	28.5
0.25	24.4

*Means and SDs over 2 positions of a typical experiment
