# Supplementary material for: Bacterial biofilm under flow: First a physical struggle to stay, then a matter of breathing
Source: PLoS One. 2017 Apr 12;12(4):e0175197. doi: 10.1371/journal.pone.0175197 (PMC5389662; doi:10.1371/journal.pone.0175197)
Supplement: S3 Table — (PDF) [file pone.0175197.s014.pdf]

S3 Table.

	Biofilm (a) versus Exponential growth phase		
	This study	Schembri et al. (b)	Beloin et al. (c)
adiC, arginine:agmatine antiporter	40,93		
**adiY, adi system transcriptional activator**	**36,46**	**13,07**	
**hyaB, hydrogenase 1 large subunit**	**36,00**	**17,15**	
hycE, hydrogenase 3 large subunit	27,02		
**hyaC, hydrogenase 1 b-type cytochrome subunit**	**26,50**	**13,99**	
**hyaA, hydrogenase 1 small subunit**	**24,28**	**65,19**	
**hycF, formate hydrogenlyase complex iron-sulfur protein**	**23,32**	**16,04**	
**gadA, glutamate decarboxylase A PLP-dependent**	**22,53**	**8,66**	**3,15**
yodB, cytochrome b561 homolog	22,53		
gadC, glutamate:gamma-aminobutyric acid antiporter	20,41		
hycD, hydrogenase 3 membrane subunit	20,35		
mdtE, anaerobic multidrug efflux transporter ArcA-regulated	20,30		
**hyaE, putative HyaA chaperone**	**19,74**	**13,47**	
**yhiM, acid resistance protein inner membrane**	**19,31**	**8,23**	
yjiY, putative transporter	18,82		
**gadB, glutamate decarboxylase B PLP-dependent**	**18,80**	**10,59**	
yhiD, putative Mg(2+) transport ATPase inner membrane protein	18,13		
**hyaD, hydrogenase 1 maturation protease**	**17,57**	**58,39**	
yhjX, pyruvate-inducible inner membrane protein putative transporter	17,19		
ykgO, RpmJ-like protein	16,69		
hycC, hydrogenase 3 membrane subunit	16,36		
dctR, Putative LuxR family repressor for dicarboxylate transport	15,83		
ykgM, 50S ribosomal protein L31 type B alternative zinc-limitation L31 protein	15,73		
**hycB, hydrogenase 3 Fe-S subunit**	**15,41**	**8,75**	
cbdB, cytochrome bd-II oxidase subunit II	15,21		
**hyaF, hydrogenase-1 protein nickel incorporation factor**	**15,02**	**21,84**	
cbdA, cytochrome bd-II oxidase subunit I	14,74		
mdtF, anaerobic multidrug efflux transporter ArcA-regulated	14,68		
**hdeD, acid-resistance membrane protein**	**14,48**	**8,25**	
hycG, hydrogenase 3 and formate hydrogenase complex HycG subunit	12,63		
hybA, hydrogenase 2 4Fe-4S ferredoxin-type component	12,56		
**hycA, regulator of the transcriptional regulator FhlA**	**11,46**	**10,83**	
gadE, gad regulon transcriptional activator	11,35		
slp, outer membrane lipoprotein	10,97		
hybO, hydrogenase 2 small subunit	10,73		
**appA, phosphoanhydride phosphorylase**	**10,68**	**10,86**	
adiA, arginine decarboxylase	10,67		
cbdX, putative cytochrome bd-II oxidase subunit	10,32		
zinT, zinc and cadmium binding protein periplasmic	10,07		

(a) This study: biofilm under flow in 250 µm height channel during 15 h; Schembri et al.: biofilm under flow during 40 h; Beloin et al.: static bioflm cultured in chemostat during 8 days

(b) Schembri et al. 2003: the authors kept only genes with Pvalue < 0.03

(c) Beloin et al. 2004: the authors just comprared expression levels
